# Radiographic and Clinical Outcomes of Laser-Enhanced Disinfection in Endodontic Therapy

**DOI:** 10.3390/jcm14124055

**Published:** 2025-06-08

**Authors:** Janos Kantor, Sorana Maria Bucur, Eugen Silviu Bud, Victor Nimigean, Ioana Maria Crișan, Mariana Păcurar

**Affiliations:** 1 Doctoral School, George Emil Palade University of Medicine, Pharmacy, Science, and Technology of Târgu Mureș, 38 Ghe. Marinescu Street, 540142 Târgu Mureș, Romania; jancsy22@gmail.com; 2 Department of Dentistry, Faculty of Medicine, Dimitrie Cantemir University of Târgu Mureș, 540545 Târgu Mureș, Romania; 3 Department of Orthodontics, Faculty of Dentistry, George Emil Palade University of Medicine, Pharmacy, Science, and Technology of Târgu Mureș, 38 Ghe. Marinescu Street, 540142 Târgu Mureș, Romania; mariana.pacurar@umfst.ro; 4 Department of Anatomy, Faculty of Dentistry, Carol Davila University of Medicine and Pharmacy, 050474 Bucharest, Romania; victor.nimigean@umfcd.ro; 5 Faculty of Medicine, George Emil Palade University of Medicine, Pharmacy, Science, and Technology of Târgu Mureș, 38 Ghe. Marinescu Street, 540142 Târgu Mureș, Romania; ioanamariacrisan16@gmail.com

**Keywords:** laser-assisted endodontics, periapical healing, cone-beam computed tomography (CBCT), bone density

## Abstract

**Background and Objectives**: Periapical healing and bone regeneration are key indicators of endodontic success. This study evaluated the effectiveness of laser-assisted disinfection compared to conventional chemical irrigation in promoting periapical healing across various bone densities (D1–D5), using cone-beam computed tomography (CBCT) over multiple follow-up intervals. **Materials and Methods**: A total of 120 patients with radiographically confirmed periapical lesions were enrolled and allocated into two groups: an experimental group (*n* = 60, chemical irrigation + Er,Cr:YSGG laser disinfection) and a control group (*n* = 60, chemical irrigation only). CBCT scans were obtained at 6 months, 1 year, 2 years, and 2.5 years post-treatment to assess lesion size and CBCT-PAI scores. Lesions were classified radiographically as either *well-defined radiolucent lesions* or *undefined* periapical radiolucencies. Paired *t*-tests and ANOVA were used for statistical comparisons. **Results**: The experimental group demonstrated significantly greater reductions in lesion size and improvements in CBCT-PAI scores at all time points. Healing was especially enhanced in low-density bone (D4–D5). Complete healing rates were higher in the laser group for *well-defined radiolucent* (89.5% vs. 68.4%) and *undefined lesions* (81.8% vs. 59.1%). Post hoc power analysis confirmed statistical reliability (Cohen’s d = 3.48; power > 0.99). **Conclusions**: Laser-assisted endodontic disinfection significantly accelerates periapical healing and promotes bone regeneration, particularly in low-density bone. CBCT imaging supports its clinical superiority over conventional irrigation methods.

## 1. Introduction

Histopathological confirmation is required for a definitive diagnosis of periapical lesions. However, as this study was retrospective, histopathological analysis was not feasible and could not be ethically or practically implemented. Accordingly, all the periapical radiolucencies were divided into *well-defined radiolucent lesions*, commonly used in the radiological literature to describe lesions with features suggestive but not confirmatory of periapical cysts [1,2], and *undefined lesions*, suggestive of chronic periapical lesions—CAP.

Endodontic treatment focuses on preserving natural teeth by eliminating infections and preventing periapical pathology [1]. Various diagnostic tools have been introduced to improve endodontic treatment [2,3], including apex locators and intraoral endoscopes to visualize internal canal structure. However, intraoral digital periapical radiographs remain the most widely used imaging modality in endodontics due to their accessibility and reliability [4]. These radiographs provide valuable insights into dentoalveolar structures, allowing clinicians to assess root morphology, canal anatomy, and treatment accuracy [2,4,5]. Nevertheless, the inconvenience of conventional periapical radiographs lies in their two-dimensional (2D) nature, which can introduce geometric distortions and limit the evaluation of lesion size, extent, and precise location [2,4,5].

However, endodontic treatment success is based on radiographic criteria. A well-sealed root canal filling and the absence of periapical radiolucency are key indicators of favorable treatment outcomes [6,7]. The length and density of the filling are crucial factors in preventing microbial infiltration and reinfection, and inadequate obturation has been strongly correlated with higher failure rates [6,7]. Follow-up radiographic examinations provide essential information on post-treatment healing, where the absence of pathological radiolucency remains a primary marker of successful therapy [8].

The primary drawback of conventional radiographic techniques is their inability to capture the three-dimensional (3D) complexity of dental structures. The limitations of 2D imaging, such as geometric distortions, magnification inconsistencies, anatomical noise, and overlapping structures, often obscure critical diagnostic details [9,10,11].

Cone-beam computed tomography (CBCT) has become a cornerstone in endodontic imaging, offering high-resolution, three-dimensional visualization of complex anatomical structures. With a spatial resolution under 0.1 mm, CBCT enhances diagnostic precision and treatment planning beyond what is achievable with traditional two-dimensional radiographs [12,13].

Widely regarded as the gold standard for hard tissue imaging, CBCT provides detailed 3D reconstructions that improve diagnostic accuracy in endodontics, periodontology, orthodontics, and maxillofacial surgery [5,14,15,16]. It enables rapid image acquisition, typically under 60 s, with significantly reduced radiation exposure (~68 µSv) compared to conventional CT (~600 µSv) [17,18,19]. Recent advances include the integration of CBCT with optical scanning for enhanced 3D modeling and treatment planning [20].

In endodontics, small field-of-view (FOV) CBCT is recommended to optimize image quality while minimizing radiation exposure, adhering to ALARA principles [14,15]. However, patient motion can affect image clarity. Studies suggest that seated or supine positioning improves stability, especially in hybrid panoramic/CBCT systems, which, despite slight compromises in image resolution, remain cost-effective and widely accessible [21,22].

Bone quality assessment is also critical for treatment planning. Using Hounsfield Units (HU), the Misch and Kircos D1–D5 classification system quantifies bone density: D1 (>1250 HU), D2 (850–1250 HU), D3 (350–850 HU), D4 (150–350 HU), and D5 (<150 HU), with distribution varying by anatomic region [23,24].

Conventional radiographs often fail to detect early periapical bone destruction due to overlapping anatomical structures [25]. In contrast, CBCT enhances detection sensitivity and reduces observer variability through the use of the CBCT Periapical Index (CBCT-PAI) developed by Estrela et al. [2,26]. This index objectively evaluates lesion size and proximity to critical structures, improving diagnostic accuracy and reliability in clinical and epidemiological contexts.

Despite these advancements, the impact of bone density on lesion healing and the potential of laser-assisted disinfection to accelerate periapical regeneration remain underexplored. Persistent intracanal infection and periapical biofilms contribute to treatment failure, resulting in chronic periapical lesions such as granulomatous or fibrotic radiolucencies [27].

Lesion healing was monitored using standardized CBCT-PAI scoring [2], offering a reproducible and clinically validated method for longitudinal assessment of periapical changes.

Specific criteria were used to ensure diagnostic accuracy and consistency in the follow-up analysis and to differentiate between *well-defined radiolucent* and *undefined lesions*. Cystic granulomas are diagnosed using radiographic and histopathological features [2,28,29,30]. Radiographically, these lesions appeared as well-defined radiolucencies with sclerotic borders, while histological examination confirmed the presence of an epithelial lining accompanied by chronic inflammatory infiltrate.

In contrast, CAP was frequently diagnosed in *undefined lesions* with persistent periapical radiolucency, lacking well-defined margins, which are cystic characteristics. These cases often included clinical signs such as tenderness, mild swelling, or sinus tract formation [26]. Histopathologically, they were characterized by granulation tissue without an epithelial component. These diagnostic distinctions were critical in establishing an accurate baseline and ensuring reliable outcome evaluation during the follow-ups [31].

Although conventional chemical irrigation with sodium hypochlorite (NaOCl) and ethylenediaminetetraacetic acid (EDTA) is the gold standard for microbial control, recent advances suggest that laser-assisted disinfection may enhance periapical healing by eliminating residual bacteria, promoting osteogenesis, and stimulating host immune responses [31].

The histopathology of periapical lesions plays a crucial role in healing dynamics. Epithelial-lined cavities tend to heal more rapidly than CAP, which consists of fibrotic connective tissue with chronic inflammatory infiltrates [32]. Additionally, bone density is a key determinant in the speed and completeness of healing, with low-density bone (D4–D5) exhibiting slower mineralization and a higher risk of persistent inflammation [33,34].

The null hypothesis (H_0_) states that there is no significant difference in periapical healing between laser-assisted disinfection and conventional chemical irrigation. The alternative hypothesis (H_1_) proposes that laser therapy enhances healing outcomes, particularly in low-density bone and *undefined lesions*.

## 2. Materials and Methods

This retrospective study evaluated the effect of laser-assisted disinfection on periapical healing following non-surgical endodontic therapy. Using cone-beam computed tomography (CBCT) at baseline, 6 months, 1 year, 2 years, and 2.5 years, this study tracked longitudinal changes in lesion resolution and bone regeneration. Serial CBCT imaging was performed using a small field-of-view and low-dose protocol, adhering to ALARA principles [27], and was approved by the institutional ethics committee. This approach enabled precise volumetric analysis of healing dynamics, particularly in bone of varying density.

A total of 120 patients with radiographically confirmed periapical lesions were included. Eligible patients had no systemic conditions affecting bone metabolism, previous root canal treatment on the affected tooth, and no contraindications to laser use. Participants were randomly assigned to two groups: a control group (*n* = 60) receiving standard irrigation with 5.25% sodium hypochlorite and 17% EDTA, and an experimental group (*n* = 60) treated with the same irrigation protocol supplemented by Er,Cr:YSGG laser disinfection.

### 2.1. Ethical Considerations

This study was approved by the Ethics Committee of the “George Emil Palade” University of Medicine, Pharmacy, Science, and Technology of Târgu Mureș (Decision No. 1885, 12 October 2022) and adhered to the ethical principles of the Declaration of Helsinki. Written informed consent was obtained from all participants.

### 2.2. Diagnostic Criteria

Due to this study’s retrospective nature, periapical lesions were diagnosed radiographically using cone-beam computed tomography (CBCT), without histological confirmation. Lesions were evaluated according to the CBCT Periapical Index (CBCT-PAI) proposed by Estrela et al. [2], considering size, location, and border characteristics. Well-defined radiolucent lesions with sclerotic margins were interpreted as suggestive of apical cysts, while *undefined lesions*, non-encapsulated radiolucencies, were classified as consistent with CAP. These radiographic distinctions were essential for accurately establishing baseline lesion status and ensuring consistent, standardized follow-up assessments. All CBCT images were reviewed by two calibrated, blinded endodontists, with disagreements resolved by a third blinded evaluator.

### 2.3. Endodontic Treatment Protocol

Multi-rooted teeth, including molars and premolars with complex canal systems, were included in this study, as these cases present greater clinical challenges and are more relevant for evaluating the efficacy of advanced disinfection methods such as laser-assisted therapy. Single-rooted anterior teeth were excluded to ensure that the study population reflected a higher level of anatomical variability, where differences in canal morphology and bacterial load are more likely to influence treatment outcomes. Only teeth with no prior history of root canal treatment were included.

The patients’ clinical records indicated they underwent standardized mechanical and chemical debridement using a crown-down technique with nickel-titanium rotary files (ProTaper Next, Dentsply Sirona, Charlotte, NC, USA). Irrigation protocols included 5.25% sodium hypochlorite (20 mL/canal) during instrumentation, 17% EDTA (5 mL/canal) as a final rinse with one-minute passive ultrasonic activation, and 2% chlorhexidine as an adjunct antimicrobial agent. Intracanal medication with calcium hydroxide paste (Ca(OH)_2_) was placed for 14 days. Root canals were obturated using the cold lateral condensation technique with Endoflas (Sanlor, Bogotá, Colombia), and the coronal access was sealed with Ketac Molar glass ionomer (3M/ESPE), followed by composite restoration using Filtek 250 (3M/ESPE) to ensure coronal integrity and prevent reinfection.

### 2.4. Laser Disinfection Protocol

In the experimental group, additional disinfection was performed with an Er,Cr:YSGG laser (Waterlase iPlus, BIOLASE, Foothill Ranch, CA, USA) set at 2780 nm wavelength, 1.5 W power, 140 µs pulse duration, and 20 Hz frequency. Laser application followed the EDTA irrigation, utilizing a radial firing tip inserted circumferentially within the canal to enhance bacterial reduction and penetration of dentinal tubules.

### 2.5. CBCT Imaging and Assessment

Periapical lesion healing was evaluated using cone-beam computed tomography (CBCT) (TVAPANO04, VATECH, Hwaseong, Republic of Korea) at predefined time points: baseline (pre-treatment), 6 months, 1 year, 2 years, and 2.5 years post-treatment. Scans were acquired using the PaX-Uni3D CBCT system with standardized parameters: 85 kV, 5 mA, and a 20-s exposure time. This setup provided high-resolution volumetric datasets optimized for the assessment of periapical pathology.

CBCT images were reconstructed and analyzed using Ez3D 2009 Plus software, version 2.1.0 (VATECH, Hwaseong, Republic of Korea). For volumetric lesion measurement, semi-automated segmentation was performed in all three orthogonal planes (axial, sagittal, and coronal), followed by manual refinement of lesion boundaries to ensure anatomical accuracy. Lesion dimensions—buccolingual, mesiodistal, and apicocoronal—were recorded, and volumetric changes over time were calculated.

Two calibrated endodontists independently performed all imaging assessments, blinded to group allocation. Inter-observer agreement was quantified using Cohen’s kappa for categorical variables (CBCT-PAI scoring, κ = 0.87) and intraclass correlation coefficients (ICC = 0.91) for continuous volumetric measurements, indicating excellent reliability. Discrepancies exceeding 10% were resolved by consensus with a third blinded evaluator.

The primary outcome measures based on CBCT included:

- Volumetric lesion size: calculated from three-dimensional measurements across buccal-lingual, mesial-distal, and coronal-apical axes.
- CBCT Periapical Index (CBCT-PAI): scored according to Estrela et al. [3] to assess lesion progression or regression.
- Bone density classification: assigned based on Misch’s D1–D5 system to evaluate healing potential relative to bone quality.

This standardized CBCT protocol allowed for accurate, reproducible, and blinded evaluation of periapical healing across all follow-up intervals.

### 2.6. Statistical Analysis

Statistical analyses were performed using SPSS version 27.0 (IBM Corp., Armonk, NY, USA), including Chi-square tests to compare categorical variables (e.g., lesion healing rates between groups) and repeated measures ANOVA to evaluate changes in lesion size and CBCT-PAI scores over time.

A significance level was set at *p* < 0.05, with *p* < 0.01 indicating a significant difference.

A post-hoc power analysis was performed to evaluate whether the sample size (*n* = 60 per group) was sufficient to detect clinically meaningful differences, particularly for the primary outcome—CBCT-PAI score at 2.5 years. Using the observed means (Experimental: 0.7 ± 0.2; Control: 1.8 ± 0.4), the pooled standard deviation was approximated as 0.316, yielding a Cohen’s d of 3.48. Based on this effect size, a two-tailed test with α = 0.05 and *n* = 60 per group achieved a statistical power > 0.99, confirming that the sample was more than adequate to detect the observed differences.

## 3. Results

A total of 120 patients (60 per group) met the inclusion criteria and completed the consent form. The experimental group received endodontic therapy with laser-assisted disinfection after EDTA irrigation, while the control group underwent conventional chemical irrigation. The patients were monitored using CBCT imaging at baseline, 6 months, 1 year, 2 years, and 2.5 years to assess changes in periapical lesion size, bone density, and CBCT-PAI scores.

### 3.1. Baseline Characteristics and Initial Lesion Distribution

Lesion Type Distribution:

- Well-defined radiolucent lesions: 76 cases (63.3%)
- Chronic apical periodontitis: 44 cases (36.7%)

Initial lesion size (mean ± SD, mm):

- Experimental group (chemical irrigation + Er,Cr:YSGG laser disinfection): 6.42 ± 1.23 mm
- Control group (chemical irrigation only): 6.51 ± 1.18 mm

Bone density distribution (D1–D5 at baseline):

- D1 (dense cortical bone): 11%
- D2 (thick trabecular bone): 21%
- D3 (intermediate trabecular bone): 28%
- D4 (sparse trabecular bone): 30%
- D5 (low density/osteoporotic bone): 10%

No statistically significant differences were found between the groups in baseline lesion size, type, or bone density distribution.

### 3.2. Lesion Size Evolution over Time

Table 1 illustrates the comparative evolution of lesion size between the experimental group (chemical irrigation + Er,Cr:YSGG laser disinfection) and the control group (chemical irrigation only) across multiple time points. There was no significant difference between the groups (*p* = 0.812) at baseline. However, from the 6-month follow-up onward, the experimental group demonstrated a significant reduction in lesion size over the control group, with statistically significant differences observed at each subsequent interval (*p* < 0.05). By 2.5 years, the experimental group achieved a mean lesion size of 0.85 ± 0.32 mm, in contrast to 1.92 ± 0.56 mm in the control group (*p* = 0.002), indicating sustained and superior long-term efficacy of the combined Laser + EDTA protocol.

### 3.3. Lesion Type and Healing Outcome

Table 2 presents the complete healing rates for different lesion types in the experimental (chemical irrigation + Er,Cr:YSGG laser disinfection) and control (chemical irrigation only) groups. Healing rates were stratified by lesion type. For well-defined radiolucent lesions, the experimental group showed a significantly higher healing rate (89.5%) compared to the control group (68.4%), with a statistically significant difference (*p* = 0.027). Similarly, in *undefined lesions*, the experimental group achieved a higher complete healing rate of 81.8%, versus 59.1% in the control group (*p* = 0.043).

### 3.4. Healing Outcome by Bone Density

Differences in healing rates were particularly evident in low-density bone (D3–D5), where the experimental protocol showed favorable outcomes.

These results suggest that bone quality influences healing potential, with the laser-assisted group demonstrating an advantage, particularly in low-density environments.

**Figure 1:**
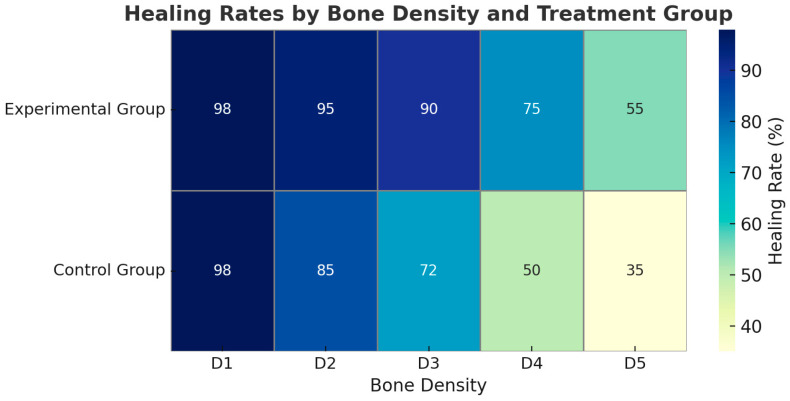
Healing Rates by Bone Density and Treatment Group.

Table 3 summarizes the relationship between bone density (classified from D1 to D5) and complete healing rates in the experimental and control groups. In high-density bone types (D1 and D2), healing rates were generally high in both groups, with no statistically significant differences (*p* = 1.00 and *p* = 0.088, respectively). However, in lower-density bone types (D3 to D5), the experimental group demonstrated significantly higher healing rates than the control group. Specifically, healing in D3 bone reached 90% in the experimental group versus 72% in the control group (*p* = 0.032), and in D5 bone, the difference was even more pronounced (55% vs. 35%, *p* = 0.009). These results indicate that the laser-assisted protocol may offer particular advantages in cases involving less dense bone structures. Figure 1 compares healing rates between the experimental and control groups in various bone density types (D1–D5).

### 3.5. CBCT-PAI Score Reduction

CBCT-PAI scores declined progressively in both groups, with statistically significant reductions observed in the experimental group at each post-treatment interval beyond baseline. Table 4 presents the evolution of CBCT-PAI scores over time in the experimental and control groups. There was no significant difference between groups (*p* = 0.76) at baseline. However, starting from the 6-month follow-up, the experimental group (chemical irrigation + Er,Cr:YSGG laser disinfection) showed more reduction in CBCT-PAI scores compared to the control group (chemical irrigation only), with all *p*-values indicating statistical significance (*p* < 0.05). By 2.5 years, the experimental group achieved a mean score of 0.7 ± 0.2, while the control group remained higher at 1.8 ± 0.4 (*p* = 0.001). These findings suggest that the laser-assisted protocol accelerates periapical healing and results in a more pronounced long-term improvement.

### 3.6. Representative Imaging and Healing Patterns

**Figure 2:**
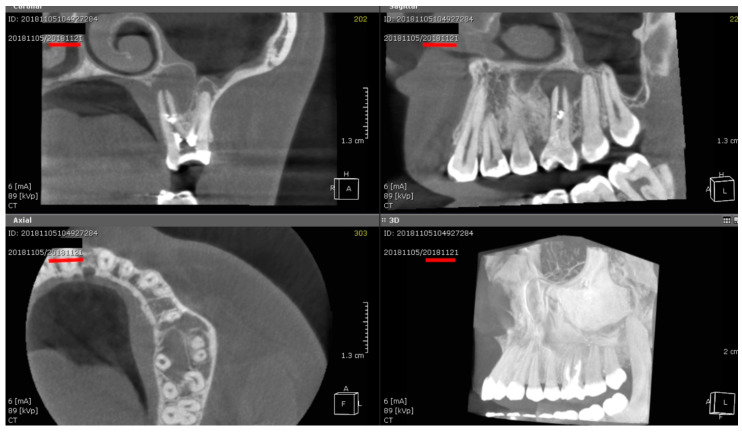
CBCT views illustrating a Maxillary well-defined lesion and Healing Outcome: coronal, sagittal, and axial slices at baseline, with 3D reconstruction showing bone regeneration at the 2-year follow-up.

Figure 2 presents CBCT images of a patient with a well-defined radiolucent lesion treated using the experimental protocol in coronal, sagittal, and axial views; it shows near-complete lesion resolution and bone regeneration at the 2-year follow-up.

**Figure 3:**
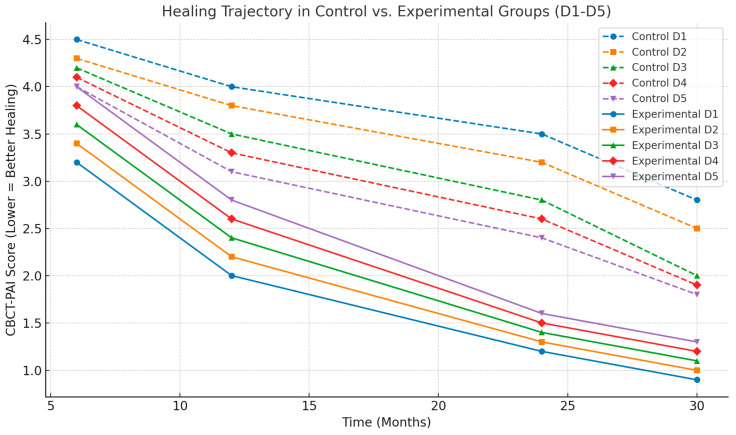
Healing trajectory in control and experimental groups, D1–D5 bone.

Figure 3 reflects the healing trajectory in the control and experimental groups over 6 months, 1 year, 2 years, and 2.5 years, considering D1–D5 bone densities. The experimental group showed a more pronounced reduction in CBCT-PAI scores, particularly in D4–D5 (lower bone density), confirming the superior effect of laser-assisted disinfection on periapical healing.

## 4. Discussion

This study evaluated the clinical impact of combining Er,Cr:YSGG laser-assisted disinfection with EDTA irrigation on periapical healing. The experimental group, treated with this combined protocol, demonstrated statistically significant improvements over the control group, which received standard chemical irrigation alone. Enhanced healing was evident through accelerated lesion resolution and greater reductions in CBCT-PAI scores, suggesting superior bone regeneration.

These findings align with previous research that highlights the limitations of conventional irrigation in fully eradicating intracanal biofilms and promoting deep tissue repair [34,35,36]. The Er,Cr:YSGG laser leverages photothermal and photoacoustic mechanisms, enabling greater penetration into dentinal tubules and more effective microbial disruption [37,38,39]. These mechanisms likely contributed to the improved clinical outcomes observed in the laser-treated group.

Laser therapy is increasingly recognized for its dual role in disinfection and biological stimulation. Its ability to promote angiogenesis, osteoblastic differentiation, and mineral deposition supports its therapeutic value in regenerative endodontics [36,37].

While both groups showed progressive healing over time, confirming the efficacy of NaOCl and EDTA irrigation [40,41], the laser-assisted group consistently outperformed the control, particularly in challenging clinical scenarios. Patients with chronic apical periodontitis (CAP) and low-density bone (D4–D5) showed markedly better outcomes in the experimental group, highlighting the adjunctive benefit of laser treatment in these conditions (Figure 2).

Only teeth without previous endodontic intervention were included to ensure uniformity in clinical response. Although microbiological analysis was not feasible due to the retrospective design, standardized treatment protocols and strict inclusion criteria minimized variability and bias.

This study also considered several contributing factors to healing, including lesion morphology, bone density, and elapsed time since obturation. A multi-timepoint analysis across 6 months, 1 year, 2 years, and 2.5 years enabled a dynamic assessment of healing trajectories across bone density categories (D1–D5). Moreover, outcomes differed between well-defined and poorly defined radiolucent lesions, suggesting that lesion characteristics and bone quality significantly affect periapical healing.

Standard irrigants—NaOCl and EDTA—are well-established for their antimicrobial and smear layer removal functions [26,27,38]. Yet, their limitations in biofilm disruption, particularly in complex canal anatomies, underscore the need for adjunctive methods [40]. Laser-assisted disinfection offers key advantages in these scenarios, including deeper tissue penetration, improved endotoxin clearance, and enhanced efficacy against resistant microbial communities [42,43,44].

The Er,Cr:YSGG laser produces photothermal and photoacoustic effects that disinfect and remove mechanical debris via cavitation and shockwave generation [43]. Unlike Low-Level Laser Therapy (LLLT), which functions primarily via photobiomodulation, the Er,Cr:YSGG system provides a high-intensity, bactericidal effect [45].

A particularly notable outcome was the superior healing of well-defined radiolucent lesions in the laser group (89.5%) compared to the control (68.4%). These results echo prior findings that lasers accelerate angiogenesis and osteogenesis in inflamed periapical tissues [40]. Likewise, CAP lesions—typically resistant to healing due to chronic inflammation and fibrotic encapsulation—responded favorably to laser treatment, with a complete healing rate of 81.8% versus 59.1% in the control group. This supports evidence that laser photobiomodulation enhances fibroblast activity, collagen remodeling, and immune response modulation [46].

Bone density played a crucial role in healing patterns. Dense bone types (D1–D2), characterized by high vascularity and robust trabecular architecture, showed strong healing regardless of group allocation, consistent with prior reports [47,48]. However, intermediate bone types (D3–D4) demonstrated a marked benefit from laser treatment, likely due to laser-induced stimulation of osteoblastic function and bone matrix deposition [49].

The most compromised outcomes occurred in D5, an osteoporotic, low-density trabecular bone with poor vascular supply and low mineral content [47]. These cases showed the slowest healing in both groups, though laser therapy still conferred a statistically significant improvement. This underscores its potential value in managing lesions in osteoporotic bone, where healing is otherwise severely limited. Studies have shown that laser energy enhances osteogenic and angiogenic pathways critical for regeneration in low-density bone [50].

Disinfection remains a cornerstone of endodontic success. In this study, the control group’s regimen of NaOCl and EDTA provided a baseline of effective cleaning [26,27] that still did not achieve the bacterial and endotoxin reduction achieved by the laser-assisted protocol. The laser produced cavitation-driven photoacoustic waves, facilitating more effective debris and biofilm removal when used with EDTA [42,43,44].

Despite the promising results, certain limitations must be acknowledged. While CBCT was instrumental in visualizing periapical healing [11,13], it cannot confirm histological changes, and its use is constrained by cost, radiation dose, and limited availability in certain healthcare environments. It is also contraindicated in specific populations such as pregnant patients [17,19].

Practical implementation of laser-assisted disinfection poses challenges. High-power laser systems like the Er,Cr:YSGG involve substantial equipment costs, maintenance demands, and specialized training [42,43,44,45], which may hinder widespread clinical adoption, particularly in low-resource settings.

The study design included blinded evaluators and demonstrated strong inter-observer reliability (κ = 0.87, ICC = 0.91), enhancing its methodological rigor. A post-hoc analysis yielded a large effect size (Cohen’s d = 3.48) and high statistical power (>0.99), confirming the sample size’s adequacy. Nonetheless, generalizability may be limited due to the relatively homogenous patient population; future multicenter trials are recommended to validate these findings across diverse demographics.

Future investigations should explore how laser therapy compares to adjunctive disinfection methods such as ozone, ultrasonic irrigation, or photodynamic therapy (PDT) [51]. In parallel, molecular research into how laser exposure modulates osteogenic and inflammatory pathways could further illuminate its regenerative mechanisms and guide parameter optimization.

## 5. Conclusions

Laser-assisted disinfection combined with EDTA irrigation significantly enhances periapical healing compared to conventional chemical methods. The laser group showed faster lesion resolution, superior bone regeneration, particularly in low-density bone, and greater reduction in CBCT-PAI scores. These findings suggest laser therapy is a valuable adjunct in endodontic treatment, especially for persistent or complex lesions. Future studies should focus on standardizing laser protocols and exploring long-term clinical outcomes.

## Figures and Tables

**Table 1:** Mean lesion size reduction (mm) over time.

Time Interval	Experimental Group	Control Group	*p*-Value
Baseline	6.42 ± 1.23	6.51 ± 1.18	0.812
6 Months	4.21 ± 0.98	5.13 ± 1.02	0.041
1 Year	2.85 ± 0.82	4.02 ± 1.01	0.008
2 Years	1.49 ± 0.64	2.83 ± 0.88	0.003
2.5 Years	0.85 ± 0.32	1.92 ± 0.56	0.002

Statistical test: repeated measures ANOVA; *p* < 0.05 considered significant.

**Table 2:** Complete healing by lesion type.

Lesion Type	Experimental Group	Control Group	*p*-Value
Well-defined radiolucent lesions	89.5% (68/76)	68.4% (52/76)	0.027
Undefined lesions	81.8% (36/44)	59.1% (26/44)	0.043

Statistical test: Chi-square; *p* < 0.05 significant.

**Table 3:** Healing Rate by Bone Density.

Bone Density	Experimental Group	Control Group	*p*-Value
D1	98%	98%	1.000
D2	95%	85%	0.088
D3	90%	72%	0.032
D4	75%	50%	0.019
D5	55%	35%	0.009

**Table 4:** Mean CBCT-PAI Score Reduction.

Time Interval	Experimental Group	Control Group	*p*-Value
Baseline	4.3 ± 0.7	4.2 ± 0.8	0.76
6 Months	3.1 ± 0.6	3.8 ± 0.7	0.041
1 Year	2.0 ± 0.5	3.1 ± 0.6	0.008
2 Years	1.1 ± 0.4	2.4 ± 0.5	0.002
2.5 Years	0.7 ± 0.2	1.8 ± 0.4	0.001

## Data Availability

The data supporting the reported results, including links to publicly archived datasets analyzed or generated during this study, can be found by emailing the corresponding author, Sorana Maria Bucur, at bucursoranamaria@gmail.com.
